# Pseudopterygium: An Algorithm Approach Based on the Current Evidence

**DOI:** 10.3390/diagnostics12081843

**Published:** 2022-07-30

**Authors:** Facundo Urbinati, Davide Borroni, Marina Rodríguez-Calvo-de-Mora, José-María Sánchez-González, María García-Lorente, Francisco Zamorano-Martín, Rahul Rachwani-Anil, Santiago Ortiz-Pérez, Vito Romano, Carlos Rocha-de-Lossada

**Affiliations:** 1 Department of Ophthalmology, Hospital Regional Universitario de Málaga, 29010 Malaga, Spain; facundou10@gmail.com (F.U.); marocalmo@gmail.com (M.R.-C.-d.-M.); glorentemaria@gmail.com (M.G.-L.); zamoranomartinfrancisco@gmail.com (F.Z.-M.); 2 Department of Doctoral Studies, Riga Stradins University, LV-1007 Riga, Latvia; info.borroni@gmail.com; 3 Advalia Vision, Cornea Research Unit, 20145 Milan, Italy; 4 Department of Physics of Condensed Matter, Optics Area, University of Seville, 41012 Seville, Spain; 5 Department of Ophthalmology, Hospital de Antequera, 29200 Malaga, Spain; rahul.medum@gmail.com; 6 Departamento de Oftalmología, Hospital Universitario Virgen de las Nieves, 18014 Granada, Spain; drsantiagoortiz@gmail.com (S.O.-P.); carlosrochadelossada5@gmail.com (C.R.-d.-L.); 7 Department of Surgery, University of Granada, 18011 Granada, Spain; 8 Department of Ophthalmology, Instituto de Investigación Biosanitaria ibs.GRANADA, 18012 Granada, Spain; 9 Department of Medical and Surgical Specialties, Radiological Sciences, and Public Health, Ophthalmology Clinic, University of Brescia, 25121 Brescia, Italy; vito.romano@gmail.com; 10 Department of Eye and Vision Science, Institute of Life Course and Medical Sciences, University of Liverpool, Liverpool L69 3BX, UK; 11 Department of Ophthalmology, Qvision, Vithas, 04120 Almería, Spain; 12 Departament os Surgery, Ophthalmology Area, University of Seville, 41012 Seville, Spain

**Keywords:** pseudopterygium, pterygium, ocular burn, ocular trauma, limbus

## Abstract

Pseudopterygium is a non-progressive conjunctival adhesion to the peripheral cornea secondary to a corneal-limbus damage. According to the literature, the main etiology is a previous eye trauma. Nevertheless, this could be biased by the existence of other underdiagnosed causes of pseudopterygium, some of which may have severe consequences for the integrity of the eye and patient’s life. This comprehensive literature review was performed based on a search on the PubMed and Google Scholar databases of relevant pseudopterygium published papers according to our current knowledge and seeks to gather the existing evidence about its diverse etiologies and clinical features, as well as to propose a diagnostic algorithm to simplify its correct approach.

## 1. Introduction

It is well known that to maintain the homeostasis and transparency of the corneal epithelium, a group of limbal stem cells are necessary, which are found in the base layer of the limbal epithelium [1]. The limbus is the transition area between the cornea and sclera. Normal limbal and limbal stem cells function as a barrier against invasion by conjunctival epithelial cells into the cornea. When damage to normal limbal stem cells and/or destruction of their niche microenvironment occurs, limbal stem cell deficiency develops, damaging the limbal barrier function and causing the corneal epithelium to be replaced with conjunctival epithelial cells, which is the hallmark of the limbal stem cell deficiency [2]. In addition, it can cause corneal neovascularization within the epithelium and superficial and deep stroma, which leads to corneal opacity and decreased vision [3].

**Figure 1:**
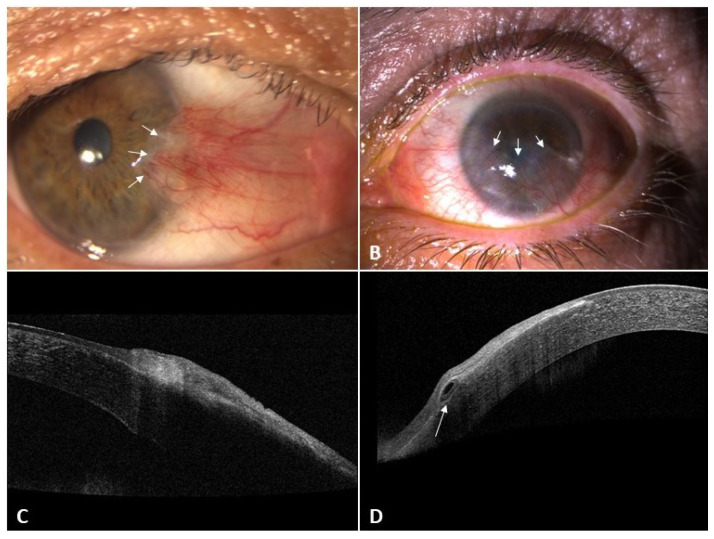
Differential features of pterygium and pseudopterygium in slit-lamp and AS-OCT examination DRI-OCT Triton Swept-Source OCT (Topcon, Tokyo, Japan). (**A**) Pterygium in slit-lamp (arrow); (**B**) pseudopterygium in slit-lamp (arrow); (**C**) pterygium in OCT (no plane of cleavage between the lesion and corneal epithelium); (**D**) pseudopterygium in AS-OCT in a horizontal scan. Notice (arrow) an overgrowing membrane that is not attached to the underlying cornea and there is a real plane of cleavage between it and the corneal epithelium. AS = Anterior segment.

Pseudopterygium is characterized by a bulbar conjunctival growth on the cornea secondary to a corneal-limbus damage. Since limbal stem cells are in charge of maintaining the homeostasis of the corneal epithelial surface, any trauma affecting them can yield profound consequences. Therefore, limbal stem cell deficiency results from an insufficient number of functional limbal stem cells caused by genetic defects, systemic immune-mediated diseases, or a secondary injury and/or insult to these cells and their microenvironment [1,4,5]. Unlike pterygium, which usually appears in the nasal section of the cornea, pseudopterygium may be present anywhere along 360 degrees of the corneal periphery [4] (Figure 1). Histologically, pseudopterygium is formed by fibrovascular tissue without the foci of elastotic degeneration which typically appears in pterygium. In pseudopterygium, the epithelium frequently extends around the fibro-vascular mass [4] and its anterior portion is usually broad and flat, as opposed to the pointed and well-defined head of the pterygium [4,6]. However, the body of the pseudopterygium may be relatively full, simulating a true pterygium [6]. 

Anterior segment optical coherence tomography (AS-OCT) is a valuable and objective method in the evaluation of conjunctivalized corneas [7]. The pterygium is sandwiched between the corneal epithelium above and a wavy destroyed Bowman’s membrane below with a mild affection of superficial stroma. Spectral domain AS-OCT images of pterygium show a wedge-shaped mass that causes an elevation of the corneal epithelium and its separation from Bowman’s layer. Immediately central to the cap of the pterygium the following structures appeared to be normal: the epithelium, Bowman’s membrane, and corneal stroma. On the other hand, in some cases of recurrent pterygium, wedge-shaped masses of tissue were found between the corneal epithelium and the underlying partly destroyed Bowman’s membrane. The central tip of these wedges was found to be more advanced and creeping beneath the corneal epithelium as opposed to what is found in primary pterygium.

In contrast, AS-OCT images in pseudopterygium show an overgrowing membrane that is not really attached to the underlying cornea. In fact, there is a real plane of cleavage between the pseudopterygium mass and the underlying corneal epithelium. Furthermore, the central point of attachment to the cornea showed invasion below the epithelium and destruction of Bowman’s membrane [8] (Figure 1). Therefore, this technology seems to be useful in the study to differentiate pterygium from pseudopterygium. However, Wang et al. reported that one limitation of AS-OCT is that the penetration is not yet deep enough in some cases. This group described the hyperreflectivity corresponding to conjunctival pseudopterygium caused signal shadowing and the impossibility of viewing of the underlying stroma. They suggested that ultrasound biomicroscopy could be useful in some of these patients [7]. On the other hand, it is necessary to remember that spectral domain ocular coherence tomography (SD-OCT) images in cases of pinguecula revealed a wedge-shaped mass that was nearly similar in pattern to pterygium but stopped at the limbal region, where there is a clearly visible line of separation between the pinguecula and the underlying scleral tissue [8].

**Figure 2:**
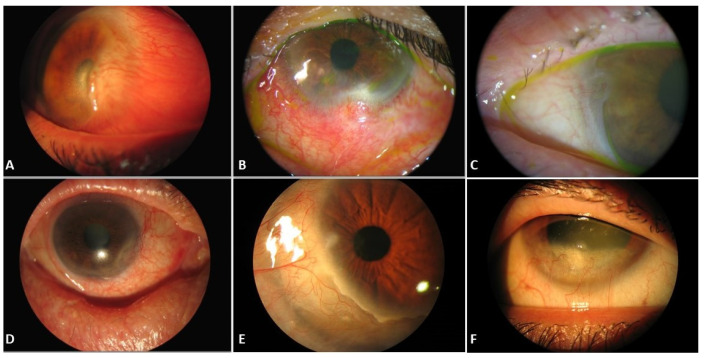
Slit-lamp images of pseudopterygium in different pathologies: (**A**) Corneal burn. (**B**) Excisional biopsy of conjunctival nevus. (**C**,**D**) Mucous membrane pemphigoid. (**E**,**F**) Terrien’s marginal degeneration.

It is known that pseudopterygium can be caused by corneal degenerations, such as marginal Terrien’s degeneration, or by corneal injuries, such as chemical or thermal burns [4]. However, there are other possible causes described in the literature, namely iatrogenic limbal damage, limbus auto-transplant, or chronic inflammation, among others (Figure 2) [4,6,8,9]. It is important to distinguish pseudopterygium from true pterygium. Firstly, the corneal thinning that often coexists with pseudopterygium can result in corneal perforation when excising the lesion [6,10]; and secondly, some of the underlying causes of pseudopterygium may lead to severe complications such as loss of globe integrity or even death [11,12,13].

To the best of our knowledge, a diagnostic algorithm for the correct characterization of pseudopterygium is not yet available in the current literature. This review attempts to place all the current available information on pseudopterygium together in one place, highlighting its diverse etiologies, its clinical features, and presenting a diagnostic algorithm to simplify its correct approach.

## 2. Materials and Methods

A review was conducted using the PubMed platform seeking prospective or retrospective review studies and case reports published until 15 February 2022, using the terms “pseudopterygium” or “pseudopterygia” in combination with keywords such as “pterygium”, “corneal degeneration”, “trauma”, “burn”, “limbus”, or “chronic inflammation”. Since the main purpose was to try to find the possible cause of pseudopterygium and not its management, we have not included articles related to the surgical technique nor described it.

Search findings:

1. Pseudopterygium: 67 articles.
2. Pseudopterygia: 9 articles.
3. ((pseudopterygium) OR (pseudopterygia)) AND (pterygium): 39 articles.
4. ((pseudopterygium) OR (pseudopterygia)) AND (corneal degeneration): 10 articles.
5. ((pseudopterygium) OR (pseudopterygia)) AND (trauma): 18 articles.
6. ((pseudopterygium) OR (pseudopterygia)) AND (burn): 12 articles.
7. ((pseudopterygium) OR (pseudopterygia)) AND (limbus): 20 articles.
8. ((pseudopterygium) OR (pseudopterygia)) AND (chronic inflammation): 2 articles.

A review of all the identified abstracts published in English and Spanish was assessed. Duplicated articles were verified including only the original version. A total of 54 unique abstracts were considered for the review; 15 articles were excluded because they were not relevant for the review, resulting in a final number of 39 articles.

All articles were read and the respective references were crossmatched to identify 8 more articles that had not been included in the initial search and were finally included in the review. Moreover, and in a similar fashion, another literature search was performed finally including 47 articles from PubMed in this study.

Later, we expanded our search using Google Scholar. A total of 466 unique abstracts were identified for inclusion in the review. We discarded articles that were not in English or Spanish and those that were not relevant for this review. Finally, 89 articles were included in the analysis.

## 3. Definition of Pseudopterygium

Pseudopterygium has been described as a non-progressive conjunctival adhesion to the peripheral cornea that may appear in any quadrant. It may be caused by trauma and/or inflammatory disease, corneal degenerations, corneal burns (thermal, chemical, or gas), post-ocular surgery with limbus involvement (iatrogenic) or traumatism, chronic inflammation (postinfectious and/or immune process), and in several ocular and systemic syndromes.

## 4. Differential Diagnosis

A true pterygium is a degenerative process that can be progressive, whereas pseudopterygium is a non-progressive consequence of trauma and/or inflammatory disease [4]. Pterygium is horizontal, most frequently nasal, and grows across the limbus invading Bowman´s layer; whereas pseudopterygium can be located anywhere, most frequently in an oblique orientation, and is not adherent to the limbus [4]. Thus, to differentiate between a pseudopterygium and a pterygium, it is important to verify whether the conjunctival neoformation is really attached to the cornea [9]. Prabhakar et al [9]. described that clinically, the ability to pass a probe beneath the apex is a useful diagnostic feature in pseudopterygium (Bowman’s probe test positive).

This peculiarity is less common in a true pterygium due to its adherence to underlying episclera and the sclera throughout the extent of the growth [9]. SD-OCT [8] may also prove that pseudopterygium is not really attached to the underlying cornea and the presence of a real plane of cleavage between the pseudopterygium mass and the corneal epithelium. Furthermore, the crucial point of attachment to the cornea showed invasion below the epithelium and above a damaged Bowman’s membrane (Table 1). 

## 5. Causes of Pseudopterygium

Corneal degenerations have been found as one of the possible causes of pterygium, among which are included Fuchs superficial marginal keratitis (FSMK) [14,15,16,17], Terrien marginal degeneration [6,18,19,20] and peripheral hypertrophic subepithelial corneal degeneration (PHSD) [21,22,23,24,25]. In other spectra of pathologies, external aggressions stand out in the form of eye burn [7,26,27,28], eye surgery with limbus involvement [20,29,30], and ocular surface traumatism [31,32], These factors produce a deficiency of limbal stem cells that could lead to the appearance of pseudopterygium. 

**Figure 3:**
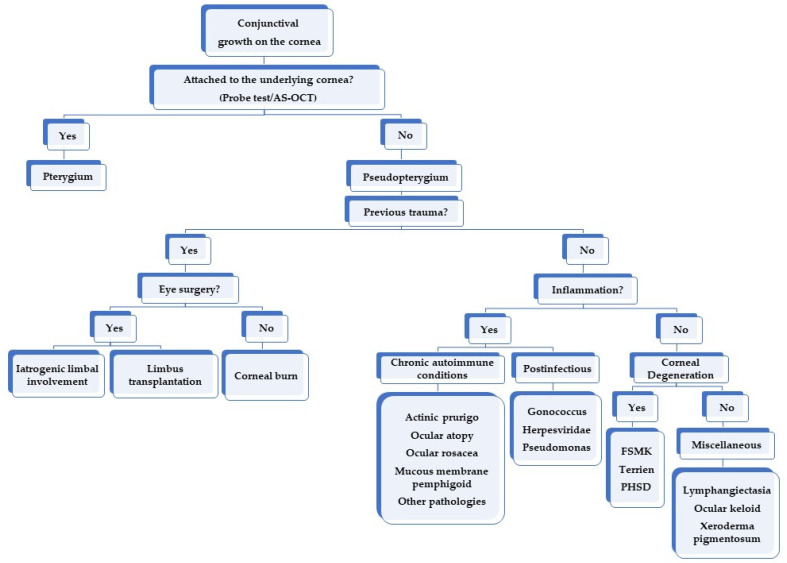
Algorithm approach to establish the etiological diagnosis of pseudopterygium. FSMK = Fuchs superficial marginal keratitis. PHSD = Peripheral hypertrophic subepithelial corneal degeneration.

Moreover, pseudopterygium could be caused by a chronic inflammatory process [33,34,35,36] such as chronic cicatrizing conjunctivitis [37,38,39], after an ocular infection due to ocular gonorrhoeae [36], herpesviridae [37], or pseudomonas [38]; due to autoimmune processes such as mucous membrane pemphigoid [12,39,40,41,42,43,44], epidermolysis bullosa [13,45], erythema multiforme [12], ocular rosacea [35,46], Still disease [18], ocular atopy [11,47], actinic prurigo [48,49,50,51,52,53], ocular psoriasis [54], or erythema elevatum diutinum [55]; and even related to ocular topical drugs [56]. Other less frequent causes with which it has been related would be Goldenhar–Gorlin syndrome [57], xeroderma pigmentosum [58], ocular keloids [59,60] and in conjunctival lymphangiectasia [61] (Figure 3).

### 5.1. Previous Eye Trauma

Homeostasis and transparency of the corneal epithelial surface are maintained by a group of adult stem cells that are located in the basal layer of limbal epithelium [1]. Limbal stem cell deficiency must be evaluated due to the possibility of destruction or dysfunction because of a corneal aggression [62]. Direct damage to limbal stem cells and the destruction of their microenvironment leads to limbal stem cell deficiency. 

As a consequence, the barrier function of the limbus is compromised, and the corneal epithelium is replaced with conjunctival epithelial cells. Eye burns [7], such as chemical [26,27,28], thermal [63], or gases [64], and previous eye surgery with limbus involvement [20,29,30] or ocular surface traumatism [31,32] could produce a deficiency of limbal stem cells that may lead to the appearance of pseudopterygium. 

#### 5.1.1. Iatrogenic Limbal Involvement

A main cause of pseudopterygium secondary to external damage includes any surgical intervention that could harm the limbus [20,29,30]. Pseudopterygium occurrence secondary to limbal surgery in limbal dermoid or cyst excision [65,66,67], excisional biopsy of giant conjunctival nevus [68], or excision of ocular surface squamous neoplasia (OSSN) has been described [69]. These procedures could lead to a limbal stem cell deficiency that could manifest in the postoperative period as a pseudopterygium extending onto the cornea. Nevertheless, theoretically it would make sense to presume that any ocular surgery with limbus involvement can cause limbal insufficiency that may promote the development of a pseudopterygium. Conjunctival and limbal-conjunctival grafts are described causes of pseudopterygium at the donor site [70]. Nevertheless, the incidence is low as reported in the literature as a result of the low amount of tissue that is necessary to be taken from the donor eye [71]. Han et al. [71] studied the efficacy and safety limbal-conjunctival autografting with limbal fixation sutures after pterygium. A total of 90 patients with pterygium and recurrent pterygium were treated with this technique. Among 103 eyes, they only reported two cases of pseudopterygium at the donor site. It is more frequent when the donor limbus is removed, probably caused by the absence of limbus which normally acts as a barrier [72].

#### 5.1.2. Ocular Burns

It is known that corneal conjunctivalization is a severe complication of limbal stem cell deficiency. Most cases are due to thermal or chemical burns [7,73]. The leading cause of pseudopterygium in an ocular burn is the destruction of corneal stem cells and the consequential persistent defects of the corneal epithelium [28]. Several articles in the literature describe cases of pseudopterygium caused by chemical, thermal, and gas burns [7,26,27,28,63,64]. For instance, Wang et al. described the clinical use of OCT in optimizing surgical treatment strategies for conjunctivalized corneas secondary to ocular burns. They studied 10 cases of pseudopterygium out of 25 patients with stable ocular burns. The thickness of the superficial conjunctivalized tissue was measured in the corneal center or 2.5 to 4.0 mm from the center. The underlying cornea with normal light reflectivity was assumed to be healthy and measured whenever possible. The cross-sectional images afforded by OCT provided useful information for making diagnosis strategies and provided guidelines for surgical treatments [7].

### 5.2. Inflammation-Related Causes

#### 5.2.1. Chronic Autoimmune Conditions

There are other well-known inflammatory diseases involving the ocular surfaces that could lead to limbal stem cell deficiency. There are several causes of pseudopterygium due to episodes of chronic inflammation reported in the literature, such as mucous membrane pemphigoid [12,39,40,41,42,43,44], epidermolysis bullosa [13,45], erythema multiforme [12], ocular rosacea [35,46], chronic blepharitis [74], Still disease [18], ocular atopy [11,47], actinic prurigo [48,49,50,51,52,53], among others. Considering these inflammatory pathologies is vital as their course can be devastating for the eye and even fatal for the patient.

Actinic prurigo is a common chronic photodermatosis that affects skin areas exposed to the sun. The involvement of the conjunctiva is common, and could provoke hyperemia, photophobia, and increased lacrimation in its initial stages. Severe conditions can lead to brown pigmentation, hypertrophy of the papillae, and pseudopterygium formation associated with pruritus [48,52].

Unlike actinic prurigo, atopy is a hypersensitivity reaction to common environmental allergens. Atopic keratoconjunctivitis (AKC) is the most severe form of actinic prurigo and it is defined as a serious, chronic external eye inflammation associated with hyperemia, conjunctival scarring, fornix shortening, and subconjunctival papillary hyperplasia [11]. The cornea and the limbus may also be affected due to the chronic inflammatory state, favoring the formation of pseudopterygium [11].

Acne rosacea is also a chronic inflammatory disease that affects the skin of the cheeks, nose, chin, forehead, and eyelids [75]. More than 50% of patients with cutaneous rosacea also suffer from ocular rosacea, which is characterized by telangiectasia, conjunctival hyperemia, meibomian gland dysfunction, sclerokeratitis, and scarring conjunctivitis. The ocular manifestations of rosacea are usually underdiagnosed by clinicians managing patients with chronic external ocular inflammation, but ocular rosacea can have a significant psychosocial impact on patients and could be a vision-threatening disease if remained untreated [75].

Ocular cicatricial pemphigoid is a chronic process and a subset of the systemic autoimmune inflammatory disease mucous membrane pemphigoid. This condition is a heterogeneous group of chronic, autoimmune subepithelial blistering diseases which predominantly involves the mucous membrane and occasionally the skin. In vivo, it is characterized by linear deposition of IgG, IgA, or C3 along the epithelial basement membrane zone [76]. Although the most common site affected is oral mucosa (85%), ocular conjunctiva is the second most prevalent at 65% [77]. It causes chronic conjunctivitis that can eventually cause progressive conjunctival scarring, leading to entropion, trichiasis, distichiasis, and lagophthalmos [40]. The deficiency of stem limbal cells and the dysfunctional tear film can lead to the formation of corneal pannus, pseudopterygium, and corneal scarring. The most severe cases can progress to corneal perforation [40].

Pseudopemphigoid, on the other hand, is usually characterized by a non-progressive unilateral scar conjunctivitis that develops in response to certain topical medications, hence it is important to rule out a mucous membrane pemphigoid [56]. There are two features that help differentiate it from mucous membrane pemphigoid: one-sidedness symptoms and non-progression of the disease after cessation of the drug. The main drugs that can cause it are timolol, pilocarpine, and latanoprost [56]. Its clinical findings are very similar to those of mucous membrane pemphigoid, with fornix shortening, symblepharon formation, trichiasis, corneal epithelial defects, entropion, corneal pannus, pseudopterygium formation, and corneal perforation [56].

Rodríguez-Ausín et al. [54] described a case series of ocular psoriasis that developed pseudopterygium after episodes of chronic blepharitis. Lekhanont et al. [55] described a case of progressive keratolysis with pseudopterygium associated with erythema elevatum diutinum, a rare, chronic, and recurrent dermatosis. 

#### 5.2.2. Postinfectious

It should be emphasized that pseudopterygium may be the clinical presentation of gonococcal conjunctivitis [36], Herpesviridae [37], or pseudomonas [38] among those described in the literature. However, it is plausible that any infection that affects the ocular surface and causes considerable damage to the limbal stem cells could cause this condition.

### 5.3. Corneal Degenerations

The most frequent corneal degenerations associated with pseudopterygium are FSMK [14,15,16,17], Terrien marginal degeneration [6,18,19,20], and PHSD [21,22,23,24,25]. 

Fuchs et al. [78] described FSMK as a marginal keratitis characterized by recurrent episodes of peripheral corneal ulceration with consequent thinning, separated from the central cornea by a gray line and often complicated with a pseudopterygium. It is an uncommon chronic disorder that presents with ocular pain, photophobia, lacrimation, and vision loss. More common in young to middle-aged adults, the process does not start from all parts of the margin at the same time [17].

Terrien et al. [79] described a bilateral, although asymmetrical, non-ulcerative disorder of the peripheral cornea that resulted in corneal thinning starting in the superior cornea and respecting the epithelium. It commonly occurs in middle-aged males and usually manifests clinically with decreased visual acuity from increased corneal astigmatism [80]. The leading edge of the lesion is delineated by a white line with calcareous deposits similar to those seen in gerontoxon, with a clear area between the opacities and the limbus. The thinning spreads circumferentially and corneal opacification and vascularization may develop over time. Terrien marginal degeneration usually progresses very slowly, sometimes over the course of 30 years [80]. Nevertheless, pseudopterygium may appear in the initial stage of the disease [6]. Goldman et al. [6] reported the characteristics of what they called atypical pterygium (namely, a pseudopterygium) in this disease located in positions other than the palpebral fissure and extending onto the cornea at an oblique angle with a broad, flat anterior edge.

PHSD is considered a possible variant of Salzmann’s nodular degeneration in the absence of chronic ocular inflammation. Although the presence of an abnormal adjacent limbal vasculature suggests chronicity, histology results do not show inflammation [21]. Actually, Salzmann’s nodular degeneration has also been suggested to be included in the differential diagnosis of pseudopterygium [61]. However, although it is possible that the pterygium associated with the Salzmann’s nodular degeneration is really a pseudopterygium similar as in PHSD, this fact is not clear according to what has been reported in the literature [25,81].

Pseudopterygium can appear in all the three entities previously mentioned, being more common in FSMK, specifically in the area of frequent keratitis where corneal thinning is more extensive. It could be explained by the inflammatory characteristic of this condition [14,15]. Although Terrien’s marginal degeneration is a slow progressive and non-inflammatory degeneration, a variant form characterized by prominent inflammation has been described. This could explain the higher number of pseudopterygium in patients with this variation [19]. Progressive corneal thinning and perforation can be seen in FSMK and in Terrien’s marginal degeneration, being less frequent in the latter [15,16,17]. These pathologies should be approached with caution in order to avoid unnecessary excision of the pseudopterygium due to the high risk of iatrogenic corneal perforation [14,19]. Kursiah et al. [20] described a case of iatrogenic corneal perforation due to the intervention of an abnormal pterygium which turned out to be a pseudopterygium in the context of a Terrien’s marginal degeneration. On the other hand, Cheung et al. [17] reported a case of a 360-degree Fuchs superficial marginal keratitis and pseudopterygium with peripheral stroma thinning that developed iatrogenic perforation at the time of superficial keratectomy.

### 5.4. Other Causes

Pseudopterygium has also been described in several other ocular surface pathologies. For instance, it has been described in a case in which an insect foreign body in the cornea induced severe marginal ulcerative keratitis [34], secondary to lymphangiectasia caused by hypodevelopment of the lymphatic system [61]. Ocular keloids are another unusual cause [59,60], defined as benign and usually single, solitary nodules that could involve the entire corneal stroma and may mimic a limbal dermoid [82]. It is not clear if the association between ocular choristomas and pseudopterygium is real [61] or it is a consequence after the excision of the former [83].

Although we did not find the term pseudopterygium in our search in some diseases, it seems logical to think that other processes, such as Stevens–Johnson syndrome/toxic epidermal necrolysis, that behave like mucous membrane pemphigoid as well as other inflammatory processes may be considered as potential causes. Similarly, ocular tumors such as OSSN [35] or sebaceous cell carcinoma could theoretically produce this condition [84,85]. In fact, Gupta et al. described two cases of OSNN in the context of xeroderma pigmentosum that developed pseudopterygium as a consequence of a presumable limbal stem cell deficiency [58]. Similarly, other dermal diseases with ocular involvement, such as dermatitis herpetiformis [86], lichen planus [87], systemic lupus and discoid lupus [88], or graft versus host disease [89] could also cause it. In addition, several ocular surface infections, such as trachoma [90], chlamydia conjunctivitis, viral keratoconjunctivitis [91], or any other microorganism that generates a great inflammation may develop progressive scarring and finally cause pseudopterygium. Therefore, in fact, it is biologically plausible that any cause of chronic inflammation could eventually lead to the formation of a pseudopterygium and should be discarded.

Based on current evidence, we have summarized our diagnosis approach for pseudopterygium in a simple algorithm mentioned in Figure 3.

## 6. Conclusions

To the best of the author’s knowledge, this if the first review that reports all the relevant causes of pseudopterygium reported in the literature. This article’s primary aim is to guide ophthalmologists to target a probable diagnosis of the underlying cause of pseudopterygium by using a proposed algorithm. It may also enable clinicians to look carefully for signs of corneal thinning or to verify whether the conjunctival neoformation is really attached to the cornea in order to differentiate both entities. Additionally, AS-OCT may be a helpful tool providing high-resolution images of the cornea and the pseudopterygium. According to the literature, the most frequent etiology reported is a previous eye trauma. However, these results may be biased because there may be other causes of misdiagnosed pseudopterygium. We consider that keeping this algorithm in mind could be especially useful in daily clinical practice. Establishing the underlying cause of pseudopterygium is important as some causes may have devastating consequences for the integrity of the eye and even the patient’s life. 

## Figures and Tables

**Table 1:** Differential diagnosis between pterygium and pseudopterygium. AS: Anterior segment.

Title	Pseudopterygium	Pterygium
Etiology	Secondary to corneal damage	Degenerative
Location	Anywhere along the 360 degrees	Nasal or temporal limbus
Condition	Stationary	Normally progressive
Form	Anterior portion broad and flat	Head pointed and well-defined
AS-OCT	Real plane of cleavage	Pseudoline of cleavage
Probe test	Positive	Negative
Histology	Fibrovascular tissue without foci of elastotic degeneration	Elastotic degeneration intermingled with epithelium and stroma

## Data Availability

Not applicable.
